# Extracellular matrix: Dystroglycan interactions—Roles for the dystrophin‐associated glycoprotein complex in skeletal tissue dynamics

**DOI:** 10.1111/iep.12525

**Published:** 2025-02-08

**Authors:** Mark Hopkinson, Andrew A. Pitsillides

**Affiliations:** ^1^ Skeletal Biology Group, Comparative Biomedical Sciences Royal Veterinary College London UK

**Keywords:** bone, cartilage, dystrophin associated glycoprotein complex, extracellular matrix, skeletal

## Abstract

Contributions made by the dystrophin‐associated glycoprotein complex (DGC) to cell–cell and cell‐extracellular matrix (ECM) interactions are vital in development, homeostasis and pathobiology. This review explores how DGC functions may extend to skeletal pathophysiology by appraising the known roles of its major ECM ligands, and likely associated DGC signalling pathways, in regulating cartilage and bone cell behaviour and emergent skeletal phenotypes. These considerations will be contextualised by highlighting the potential of studies into the role of the DGC in isolated chondrocytes, osteoblasts and osteoclasts, and by fuller deliberation of skeletal phenotypes that may emerge in very young mice lacking vital, yet diverse core elements of the DGC. Our review points to roles for individual DGC components—including the glycosylation of dystroglycan itself—beyond the establishment of membrane stability which clearly accounts for severe muscle phenotypes in muscular dystrophy. It implies that the short stature, low bone mineral density, poor bone health and greater fracture risk in these patients, which has been attributed due to primary deficiencies in muscle‐evoked skeletal loading, may instead arise due to primary roles for the DGC in controlling skeletal tissue (re)modelling.

## 1 INTRODUCTION

Coordinated cell–cell and cell‐extracellular matrix (ECM) interactions contribute fundamentally to tissue development, structure, homeostasis and pathobiology. The dystrophin‐associated glycoprotein complex (DGC), which serves as one mediator of such cell‐ECM interactions, is a multimolecular transmembrane assembly, linking the internal cell actin cytoskeleton to the ECM. The spanning of the cell cytoskeleton‐ECM interface by the DGC relies on dystrophin, which binds via its N‐terminus to the actin cytoskeleton, intracellularly, and to the transmembrane β‐dystroglycan protein at its C‐terminus; non‐covalent linkage of β‐dystroglycan to α‐dystroglycan, in turn allows for binding to ECM proteins containing laminin globular (LG) domains, such as laminin, neurexin and agrin in a calcium‐dependent manner.^1^

This review will share details of the cellular components and functions of the DGC, based mostly upon extensive research into its role in membrane stability in muscular tissues, and will provide an overview that extends to functions in non‐muscle tissues with a detailed focus on skeletal health. We will therefore include consideration of the roles of the major extracellular DGC ligands in controlling skeletal cell function and will culminate in an exploration of related adhesions, both focal and podosomal, and downstream signalling from the DGC with view to their possible repercussions for skeletal tissue homeostasis. Some of these considerations will, additionally, be informed both by our own recent studies exploring the role of the DGC in isolated skeletal cells, namely chondrocytes, osteoblasts and osteoclasts, and by our discovery of new skeletal phenotypes in mice lacking vital, yet diverse core elements of the DGC.

## 2 THE DYSTROPHIN‐ASSOCIATED GLYCOPROTEIN COMPLEX (DGC)

Dystroglycan is an essential component of the DGC and has most commonly been considered in the context of its roles within skeletal muscle. This is despite findings that demonstrate that it has wide distribution in a variety of tissues.^2^ Dystroglycan has indeed emerged as an important receptor for cell‐ECM interactions in many non‐muscle tissues, including brain,^3^ kidney,^4^ eyes^5^ and heart.^6^ Dystroglycan is also known to serve important roles in multiple cellular and tissue processes, such as embryonic development,^7^ epithelial morphogenesis,^8^, ^9^ cell adhesion and migration,^10^ and cell polarisation^11^ as well as being critical for normal muscle function.

The role of dystroglycan as a cell adhesion molecule is thought to encompass the fulfilment of two main functions. Firstly, it functions to provide a physical connection between cells and their ECM: anchoring the cell to the pericellular matrix, which is integral to membrane stability and in protecting against mechanically induced damage during, for example, the contraction in skeletal muscle. Secondly, dystroglycan serves direct roles as a transducer of signals from the external cellular milieu to the inside of the cell.^12^ It is salient to emphasise that the DGC has, however, also been shown to act as a signalling scaffold for pathways that do not directly involve dystroglycan‐mediated ECM binding. These pathways include signalling mediated via nitric oxide synthase,^13^ phosphoinositide 3‐kinase (PI3‐kinase^14^) and protein kinase B (Akt^15^) as well as pathways involving G‐proteins, mediated through dystrophin, dystrobrevin and syntrophins.^16^

Alpha‐dystroglycan (α‐dystroglycan) contains a heavily glycosylated mucin‐like central region that joins the N‐ and C‐terminal domains.^17^ This central region binds to a globular domain^18^ within skeletal muscle laminin‐211 and also laminin‐111^19^ expressed in non‐muscle tissues. Glycosylation of this mucin‐like region also confers the capacity for binding to a multitude of other ECM ligands, including agrin,^20^ perlecan,^21^ neurexin^22^ and laminin; the latter is required for basement membrane assembly^23^ and is essential in development of Reichert's extra‐embryonic membrane.^7^ The vital role of this glycosylation is emphasised by mutations in glycosylating factors of α‐dystroglycan, such as fukutin, fukutin‐related protein (FKRP), protein O‐linked mannose *N*‐acetylglucosaminyltransferase 1/2 (POMNT1/2) and LARGE xylosyl‐ and glucuronyltransferase 1 (LARGE), which result in defective glycosylation of the mucin‐like region of α‐dystroglycan, severely affecting its ECM binding, and hence functionality.^18^ Together, these data suggest that the roles of individual components of the DGC, as well as this vital glycosylation of dystroglycan itself, extend to non‐muscle tissues and beyond functioning purely for the establishment of membrane stability.

Focus upon the role of these DGC–ECM interactions in muscle is understandable, due to the fact that mutations, affecting DGC structure or in factors controlling the vital glycosylation of α‐dystroglycan, clearly account for the most severe forms of muscular dystrophy. Duchenne muscular dystrophy (DMD), the most common muscular dystrophy affects 1 : 7250 males aged 5–24 years,^24^ is an X‐linked recessive disease where dystrophin gene mutations result in sarcolemma instability and progressive muscle damage and weakness.^25^ The impact of mutations upon DGC function is further emphasised in the classification of a family of secondary dystroglycanopathies, which are a heterogenous group of muscular dystrophies characterised by the defective glycosylation of α‐dystroglycan.^26^ Currently, mutations in 18 genes are known to produce such secondary dystroglycanopathies; the most common are in FKRP,^27^ which are linked with a wide clinical disease spectrum, ranging from severe Walker–Warburg syndrome to milder adult‐onset limb‐girdle muscular dystrophy (LGMD2I).^28^, ^29^, ^30^, ^31^ This focus on muscle may, however, have somewhat undermined a range of potentially important DGC functions in non‐muscular tissues.

Despite the clear clinical focus on the defects in skeletal muscle function that develop due to these DGC mutations, poor bone health is indeed also common in muscular dystrophy patients. Impact on the skeleton includes the emergence of short stature,^32^ low bone mineral density, osteomalacia, osteoporosis and osteopenia^33^ with resultant increases in fracture risk.^34^ Here, we emphasise the need to better understand the aetiology of these skeletal manifestations of defective DGC function, which have historically been attributed, almost exclusively to reduced bone loading due to primary deficiencies in the strength of muscle contractions.^35^ Their aetiology may also have likely been complicated by other pathophysiological disease factors, and possible side‐effects of the anti‐inflammatory glucocorticoid therapy that muscular dystrophy patients commonly receive.

Although many murine muscular dystrophy models exist, most research into the role of the DGC in skeletal health has focused on human DMD patients, with only a few studies reporting upon skeletal characteristics in mouse disease models^36^; skeletal data for other muscular dystrophy subtypes that affect DGC components are scarce. Studies in the mdx mouse DMD model have previously observed skeletal tissue differences, yet nearly all were performed after the onset of muscle damage^37^, ^38^, ^39^; this relatively late tissue sampling made it impossible to discern any possible direct roles for the DGC in the cell‐ECM interactions that are important in skeletal function. One study by Nakagaki et al.,^40^ however, importantly observed shorter femora, lower bone volume and mineral density and impaired collagen organisation culminating in weaker bones in mdx mice (vs. wild type) at an age prior to the onset of any overt muscle damage. Interestingly, Gao et al.^41^ also reported early modifications in the femurs of 5‐day‐old mdx mice, but in contrast described significantly higher cortical bone thickness which coincided with mild muscle pathological necrosis and central nucleated fibres. Wood et al. also observed a higher cortical bone fraction in both the mdx and in the mdx:Cmah^−/−^ mouse, which harbours a human‐like mutation in the cytidine monophospho‐*N*‐acetylneuraminic acid hydroxylase gene, at 7 weeks of age. This mdx/Cmah^−/−^ mouse model is considered to more closely mirror the phenotypic and molecular signatures of human DMD (vs. mdx). It is therefore compelling that these mutant mice have also been reported to exhibit an accelerated longitudinal bone growth rate even in the presence of only a very mild muscle pathology.^42^ Together, these modifications in skeletal phenotype prior to the emergence of any profound muscle pathology in murine muscular dystrophy models suggest that they may not, necessarily, be purely a product of deficiencies in the strength of muscle contractions and the secondary reduction in loading‐derived anabolic/anti‐resorptive bone stimulation. *Do these findings instead imply that the DGC has a primary role in controlling the (re)modelling of skeletal tissues?*

This review will address this question by describing aspects of expression and functionality of extracellular DGC binding partners, intracellular DGC components and the likely downstream signalling evoked by DGC engagement. This will be completed with view to resolving α‐dystroglycan and DGC function in non‐muscle tissues and will be completed with a particular focus on cartilage and bone cells that may contribute to the emergence of skeletal phenotype.

## 3 ROLES OF THE MAJOR EXTRACELLULAR DYSTROGLYCAN LIGANDS IN THE SKELETON

We review how three major DGC‐linked ECM proteins—for which roles in cartilage and bone are defined—appear to control the behaviour of the relevant cell types to account for shifts in skeletal phenotype. It is pertinent, however, to stress that the heavily glycosylated α‐dystroglycan component of the DGC can indeed bind to vast range of ECM proteins to control cell behaviour, and that these specific ECM ligands chosen here could also serve roles that are independent of the DGC.

### 3.1 Agrin

An important DGC–ECM interaction in skeletal muscle occurs at the neuromuscular junction, where dystroglycan binds an ECM heparan sulphate‐containing proteoglycan ligand, agrin, to induce acetylcholine receptor clustering.^43^ Importantly, an agrin splice variant that lacks this receptor clustering function has also been found in a number of non‐muscle tissues, including kidney,^44^ heart^45^ and lung.^46^ In these tissues, the C‐terminal laminin globular domains of agrin bind to the mucin‐like domain of α‐dystroglycan, and its N‐terminal region binds to laminin.^47^ Beyond skeletal muscle, agrin: dystroglycan interaction is found to be vital in development and function of the heart, promoting cardiomyocyte proliferation via Yap‐ and ERK‐mediated signalling.^45^

**FIGURE 1:**
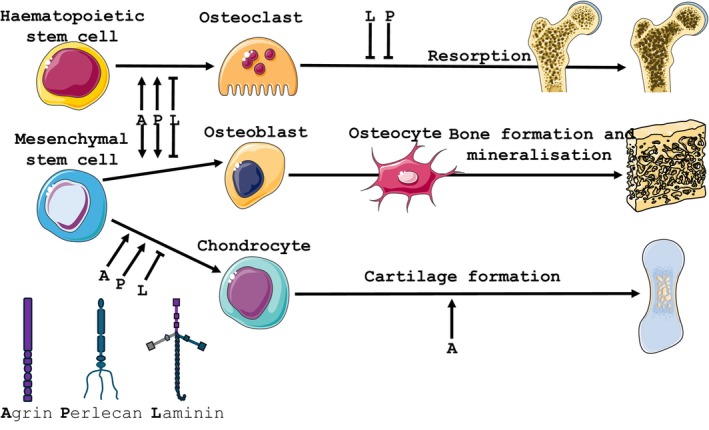
Proposed roles for laminin globular (LG)‐domain‐containing extracellular matrix (ECM) proteins, agrin (A), perlecan (P) and laminin (L) in skeletal cells and tissues. Schematic model of proposed roles of agrin, perlecan and laminin in osteoclastogenesis, osteoblastogenesis, chondrogenesis, bone resorption and cartilage formation.

These non‐muscle tissue roles of agrin are now known to extend to skeletal tissues (Figure 1). Studies by Hausser et al.^47^ using agrin‐deficient mice established that agrin was highly expressed by chondrocytes predominantly in proliferating and prehypertrophic growth plate zones where it serves critical roles in normal longitudinal growth by contributing to the essential collagen type II and aggrecan deposition as well as to proliferation. These findings were extended by the elegant work of Eldridge et al.^48^ in which agrin interactions with α‐dystroglycan and the co‐receptor LRP4 were found to drive in vitro chondrocyte differentiation and cartilage formation. These authors later demonstrated that a single intraarticular administration of agrin in a collagen type I‐containing gel was sufficient to promote cartilage regeneration.^49^

Recent studies have also identified agrin as an osteoblast regulator. Evidence shows increased agrin, dystroglycan and Lrp4 expression during osteoblast differentiation, and data from agrin‐knockdown support a reduced expression of these dystroglycan/Lrp4 receptors and restricted osteoblast differentiation via an impairment in both Wnt and BMP signalling.^50^ Osteoblast agrin expression is also known to play a critical role in the haematopoietic niche, where agrin‐expressing endosteal bone lining mesenchymal stem cells and osteoblasts were found to interact directly via α‐dystroglycan with haematopoietic stem cells. Using competitive reconstitution assays to assess sustained haematopoietic capacity, it was found that the α‐dystroglycan blocking antibody, IIH6, reduced reconstitution, thus confirming its function as a receptor ensuring haematopoietic stem cell survival and proliferation via binding to agrin expressed by stromal cells.^51^ This role for agrin in stem cell survival was further supported by data from agrin‐deficient mice where osteoclast number was accordingly also reduced.^52^ These studies suggest that agrin supports chondrocyte and osteoblast differentiation and drives osteoclastogenesis consistent with direct roles for agrin: dystroglycan interaction in the development and maintenance of both cartilage and bone skeletal tissues (Figure 1).

### 3.2 Laminin

Alpha‐dystroglycan ligands, laminin and perlecan, are also expressed in the pericellular ECM of chondrocytes where they are thought to serve as a functional equivalent to a basement membrane‐like structure.^53^ These pericellular regions around chondrocytes comprise higher proteoglycan concentrations, contain a finer arrangement of collagen characterised by the presence of collagen type VI fibres and are considered dissimilar from the more distant territorial and inter‐territorial ECMs. Elegant studies have disclosed that these laminin‐rich pericellular regions within cartilage serve roles in transducing both biochemical and biomechanical signals.^54^

Laminins are most often considered as a major basement membrane component. Family members are heterotrimeric glycoproteins, comprised of α, β and γ subunit chains with five, three and three genes encoding each respectively.^55^ The subunits can be assembled into 16 different heterotrimers, with splice variants included, some of which exhibit tissue specificity, and some which are developmentally regulated.^56^ Laminin: α‐dystroglycan binding is required for multiple cellular processes, including polarisation,^11^ adhesion^57^ and migration.^58^ α‐dystroglycan binds the alpha 1, 2, 4 and 5 laminin chains^59^, ^60^; in adult skeletal muscle, it binds to the most abundant isoform, laminin‐211 to provide membrane stability and thus ensures muscle integrity.^61^ Beyond muscle, α‐dystroglycan is known to bind other isoforms, including laminin‐511 in clonal human induced pluripotent stem cell lines^62^ and laminin‐111 in kidney, mammary gland^63^ and lung where it is integral for branching epithelial morphogenesis.^64^

Laminin is also important during cartilage formation.^65^ Kvist et al.^53^ describe varying spatiotemporal expression patterns for α1, α5, β1 and γ1 laminin subunits, which were all progressively redistributed from a diffuse‐to‐pericellular localisation during articular cartilage development. Distinct laminin chain expression patterns were also observed in intervertebral disc development, where a similar diffuse‐to‐pericellular redistribution was again observed.^66^, ^67^ Laminin has also been shown to be an important component of septal cartilage^68^ and menisci,^69^ where expression was found to be significantly diminished in degenerative, diseased samples^69^, ^70^ as well as in samples from ageing individuals.^71^ These findings suggest that laminin serves important roles in the developmental emergence of cartilage and that laminin‐related shifts in expression may be indicative, or causative of cartilage functional failure.

These links are reinforced by data indicating that interactions with exogenous laminin or laminin‐derived peptides serve to regulate articular chondrocyte ECM attachment and migration.^72^ Laminin α4 is also known to promote cell migration, with α4‐selective in vitro blockade engendering a reduction in chondrocyte clustering.^73^ Such regulatory roles for laminin in cell migration have been shown to be mediated via its interaction with α‐dystroglycan in endothelial cells^19^, ^58^ and myoblasts.^19^ Co‐expression of α‐dystroglycan and laminin in cartilage support roles for this interaction in controlling chondrocyte migration in both health and disease. Chondrogenic roles for laminin were further strengthened by the studies of Schminke et al.^74^ in which it was demonstrated that exogenous laminin treatment was capable of enhancing collagen II, whilst supressing collagen I mRNA levels in healthy and OA cartilage, and in chondroprogenitor cells. In contrast, Hashimoto et al.^75^ found that laminin suppressed chondrogenic differentiation in the ATDC5 cell line. Whilst these divergent data may be explained in the contrasting use of cell lines and primary cells, they nonetheless support roles for laminin: α‐dystroglycan in regulating chondrocyte behaviour (Figure 1).

Interestingly, laminin 5 alone, expressed by human mesenchymal stem cells, has been shown to stimulate gene expression patterns and ECM production associated with the osteogenic phenotype.^76^ Indeed, Vukicevic et al.^77^ demonstrated that discrete domains of the laminin protein can promote osteoblastic differentiation and development of osteocyte‐like canalicular processes in primary cells and the MC3T3‐E1 cell line. Laminin also appears to switch human dental follicle cell behaviour to restrict expression of early osteogenic markers and alkaline phosphatase activity in response to osteogenic induction of FAK/ERK pathway activation.^78^

Laminin expression in osteoblasts also, intriguingly, serves to negatively regulate osteoclastogenesis. Thus, coating of cell culture dishes with laminin‐332—which primary osteoblasts were found to express—was shown to inhibit osteoclast differentiation and RANKL‐induced mitogen‐activated protein kinase activation in bone marrow‐derived macrophages that had subsequently been seeded on these coated dishes.^79^ Osteoclast precursor adhesion and migration, processes integral to full differentiation, have also been shown to be induced via selective laminin binding.^80^ Together, these findings may have important implications in the control of bone remodelling, suggesting that the engagement of ECM laminin by the DGC may promote osteogenesis and limit bone resorption.

In this regard, it is important to also consider roles for laminin in osteocytes, where adhesion to their local ECM is considered integral to their essential mechanosensing function that impacts pointedly to bone homeostasis, through the downstream regulation of osteoblast and osteoclast behaviours. Osteocytes are also capable of binding to laminin, with in vitro culture of chick primary osteocytes demonstrating that the range of ECM proteins bound was analogous to those bound by osteoblasts, despite the contact areas for the former being markedly smaller.^81^ Recent studies demonstrated that a laminin 111, E8 fragment, conjugated to the growth‐factor‐binding domain of the DGC ligand, perlecan, was capable of inducing pluripotent stem cells into skin‐derived precursor cells which could then differentiate into osteocytes.^82^

Dystrophin‐associated glycoprotein complex‐mediated cell linkage to the ECM is often disrupted in muscular dystrophy, with deficient interaction between laminin and α‐dystroglycan comprising an essential characteristic of secondary dystroglycanopathies. Loss of this interaction results in membrane instability and muscle necrosis; however, it also disrupts downstream signalling, including the PI3K/AKT pathway. Blockade of laminin: α‐dystroglycan binding in primary mouse myoblast cultures using the IIH6 antibody can indeed inhibit AKT activation to reduce antiapoptotic signalling, and hence promote cell death.^15^ Interestingly, mdx mice that possess reduced amounts of α‐dystroglycan showed elevated levels of AKT activation and integrin beta 1 expression: the latter binds laminin, potentially compensating for the loss of dystroglycan‐laminin binding.^14^ The studies of Peter et al.^83^ showed that myogenic AKT signalling could also increase utrophin expression to, likewise, compensate for such loss of dystrophin, thus serving to increase membrane stability. Dystroglycan binding to laminin has also been shown to supress ERK activation induced by in vitro integrin engagement.^84^

These data indicate that direct binding of laminin by the DGC serves key roles in controlling cell behaviours and signalling pathway activation for important developmental and homeostatic functions in skeletal cell types (Figure 1), which likely contribute to net bone accrual. Whilst it is established that the dysregulation of this laminin‐dependent DGC‐mediated control contributes to muscle pathology, the full extent to which it may also contribute to pathological skeletal phenotypes linked with muscular dystrophy is only ill‐defined.

### 3.3 Perlecan

Perlecan is a large heparin sulphate‐rich proteoglycan (HSPG) which adopts an HS/chondroitin sulphate (CS) hybrid form in most tissues, with HS substitution by CS at one‐third of glycosaminoglycan binding sites near the N‐terminus.^85^, ^86^ Perlecan is another major component of basement membranes which has a wide distribution in various tissues throughout development and into adulthood, including brain, lung, liver, kidney, heart, blood vessels, intestines and the skeletal system.^87^ Perlecan has a multitude of roles within these tissues; in embryo development, it acts as an extracellular scaffold modulating signalling pathway activation, aiding cell attachment and recruitment of growth factors to promote differentiation and proliferation,^88^ as well as providing basement membrane stability^89^ and regulating cell migration and proliferation.^90^

Perlecan is a modular multifunctional HSPG with five distinct functional domains.^91^ The C‐terminal domain‐V bears three laminin‐type globular (LG) domains,^92^ which facilitate strong binding to α‐dystroglycan.^60^, ^93^ In muscle, perlecan plays an integral role in neuromuscular junction formation where it interacts with α‐dystroglycan to form a receptor for acetylcholinesterase clustering.^94^ Perlecan knockdown in zebrafish results in a severe myopathy characterised by significant loss of myofilaments with aberrant organisation and disrupted mitochondria.^95^

Perlecan is also, however, present in cartilaginous tissues,^85^ localising mostly to the pericellular ECM of chondrocytes.^53^ Its importance during skeletal development is evident from the abnormalities that are observed in perlecan‐null mice. Here, perlecan knockout was achieved by deletion of exon 7 of the Hspg2 gene, which induces 40% lethality at E10.5, with the remainder undergoing perinatal death. Those surviving to birth exhibit skeletal abnormalities with 80% displaying disproportionate dwarfism with large domed skulls and craniofacial abnormalities. Vertebral bodies were shorter and wider with multiple ossification centres, and fore and hindlimbs were bowed, shorter and broader. Growth plate organisation was severely disrupted with reduced proliferative activity and columnar structures of hypertrophic chondrocytes were lacking as was the hypertrophic marker, Col10a1.^96^ These data reveal critical roles for perlecan in the endochondral ossification processes in the skeleton (Figure 1).

Perlecan gene mutations in humans are also known to cause skeletal disorders, including Schwartz‐Jampel syndrome (SJS) and extremely rare neonatal lethal dys‐segmental dysplasia, Silverman‐Handmaker type (DDSH).^97^ DDSH patients exhibit an absence of perlecan^98^ and varying degrees of myotonia and chondrodysplasia, whilst those with SJS deposit partially functional forms of perlecan, containing a defective domain V, into the cartilage ECM and exhibit multiple, early‐onset, skeletal abnormalities, including short stature and milder osteo‐chondrodysplasias.^97^ Mouse SJS models also display similar skeletal defects; perlecan knock‐in mice possessing a C‐to‐Y mutation at residue 1532 also exhibited reduced perlecan secretion linked with smaller size, less mineralisation, misshapen bones and joint dysplasias.^99^ Xu et al.^100^ showed that this mutation resulted in chondrocyte pericellular ECM disorganisation, with lower cell and matrix stiffness in developing cartilage. These studies place perlecan and its potential to bind α‐dystroglycan centre stage in control of endochondral ossification processes in the cranium and in normal growth plate function and longitudinal growth regulation in the appendicular skeleton.

Cartilage perlecan content is also essential for vascular invasion during endochondral ossification, with defined in vitro roles in enhancing endothelial cell VEGF/VEGFR signalling. Perlecan‐null mice thus exhibit abnormal bone development with shorter, wider femora possessing a complete absence of bone marrow and trabecular bone due to a lack of vascular invasion and subsequent cartilage remodelling or bone formation by osteoblasts.^101^ Further studies using hypomorphic mutant mice that secrete negligible amounts of perlecan in skeletal tissues revealed a similar phenotype, with shorter, wider bones attributed to abnormal growth plate organisation with decreased chondrocyte proliferation and increased terminal differentiation and calcification. In vitro differentiation assays using primary embryonic cells from the hypomorphic mutants showed that the lack of perlecan secretion resulted in enhanced osteoblastogenesis, which was more pronounced in the presence of BMP2, suggesting the absence of perlecan promotes BMP2 activity and osteoblast differentiation.^102^ DeCarlo et al.^103^ observed the strong interaction between perlecan domain 1 and BMP‐2 in vitro and that during in vivo de novo bone formation on a scaffold, perlecan significantly improved dose effectiveness of BMP‐2 osteogenic activity.

Migration and proliferation of osteoblasts and chondrocytes is also modified by perlecan (Figure 1); exogenous perlecan inhibits primary osteoblast and chondrocyte migration, without affecting proliferation. However, in high density cultures, antiserum‐induced blockade of endogenous perlecan resulted in reduced proliferation of chondrocytes and osteoblasts.^90^ The lack of vascular invasion of the growth plate in perlecan‐null mice also led to reduced osteoclast numbers, differentiation (TRAP‐positive cells) and multinucleation.^101^ In contrast, there were 20% more osteoclasts observed in the proximal tibial growth plate of newborn long bones in the murine SJS model (vs. wild type^99^). Parajuli et al. found that adult SJS‐mimicking, perlecan hypomorphic mice exhibited reduced distal femur and vertebral trabecular bone mass that was linked with increased osteoclast numbers; in vitro studies verified greater osteoclastogenesis and resorption in cells from these hypomorphic mice (vs. WT^104^). These data indicate that perlecan interaction with α‐dystroglycan may profoundly regulate both cartilage and bone cell behaviour and hence contribute to the control of bone (re)modelling, bone mass and architecture during growth and maturation.

Perlecan is also a major component of the osteocyte pericellular ECM (PCM), where it serves an essential mechanosensory role, ensuring integrity of the osteocyte lacunocanalicular system that regulates bone development and turnover.^91^ Electron microscopy in perlecan‐null mouse bones showed a reduced canalicular pericellular area, density, with fewer canalicular tethering elements.^105^ Interestingly, perlecan‐poor hypomorphic mice also exhibited greater solute transport, PCM fibre density and higher bone shear stresses and were, intriguingly, non‐responsive to tibial loading; supporting a vital role for osteocyte perlecan in mechanoadaptive bone (re)modelling.^106^ This is further reinforced by defective strain‐induced calcium signalling and RNA sequencing data in tibiae; the latter show suppressed calcium signalling, ECM–receptor interaction and focal‐adhesion pathway activation in these hypomorphic mice.^107^ These studies together suggest that disrupted interaction of perlecan domain V with the α‐dystroglycan LG‐binding domain leads to aberrant cartilage and bone development and homeostasis, and ultimately the skeletal disorders linked to SJS and DDSH but also those associated with muscular dystrophy. This, additionally, points to significant roles for perlecan: α‐dystroglycan engagement in the attainment of a normal skeletal phenotype.

Our combined evaluation of the likely roles for these major DGC ligands—agrin, laminin and perlecan—in the skeleton introduces an alternative ‘skeletocentric’ perspective to the skeletal manifestations of muscular dystrophy. This places primary roles for DGC in the skeleton and differs markedly from the ‘musculocentric’ perspective, in which *all* skeletal traits in patients are assigned as secondary, due to reduced muscle activity. This view also places DGC: ECM interactions central to regulating chondrocyte, osteoblast, osteoclast and osteocyte behaviour (Figure 1).

## 4 SIGNALLING IN DGC FUNCTION

Dystrophin‐associated glycoprotein complex skeletal roles may likewise be revealed by examining the downstream cell signalling evoked in response to its variable ECM interaction and diverse dystrophin‐mediated intracellular connections. Here, we explore pathways involved in DGC function with known roles in skeletogenesis and turnover: Notch, Hippo‐YAP/TAZ, PI3K/AKT, ERK–MAPK and Src signalling. This is undertaken in the likelihood that it may clarify whether the *skeletocentric* view of the phenotypes seen in patients and mouse models of muscular dystrophy has merit, even though each of these pathways is clearly activated via a vast range of alternative stimuli. It is vital, nonetheless, to acknowledge this to be a potentially over‐simplified perspective: for example, it is clear that distinct dystrophin isoforms exhibit cell type‐specific expression^108^, ^109^, ^110^ to influence DGC intracellular interactions and downstream cell signalling and behaviours and that the loss of full‐length dystrophin in mdx mice results in downregulation of dystroglycan^111^ which may, in turn, control downstream signalling.

### 4.1 Notch signalling

Notch signalling is highly conserved and influences a wide range of tissues, including: skin, liver, testis, skeletal muscle and cartilage.^112^ It is pre‐eminent in embryonic cell fate determination and serves roles in maintaining stem cells, regulating differentiation and proliferation.^113^ Early work identified the Notch pathway as a hub for direct cell–cell signalling interactions in developmental processes; in mammals, this involves engagement of Notch receptors (Notch1‐4) and Notch ligands (Jagged1/2, Dll1, Dll3 and Dll4^114^). More recently, Notch signalling has however been found to function much broadly as an integrator of microenvironmental cues that encompass ECM ligands, mechanical shear stress, hypoxia, hyperglycaemia and other factors.^115^

**FIGURE 2:**
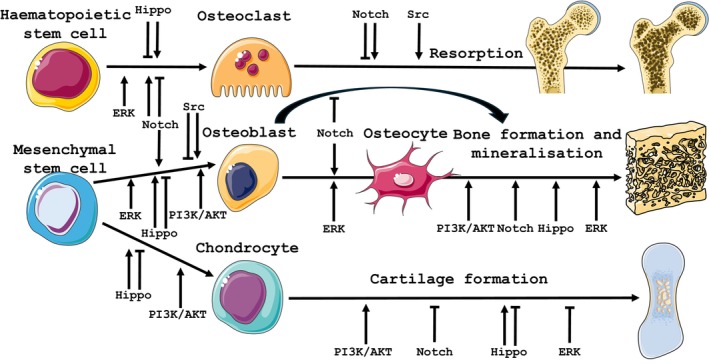
Proposed roles for dystroglycan‐associated signalling pathways in skeletal cells and tissues. Schematic model of proposed roles for Notch, Hippo, ERK, PI3K/AKT and Src signalling in osteoclastogenesis, osteoblastogenesis, chondrogenesis, bone resorption/formation and cartilage formation.

Dystroglycan is recognised as a key downstream component controlled by the Notch pathway^116^; with Notch pathway activation/inhibition found to increase/decrease the number of dystroglycan‐expressing cells in Xenopus embryos.^116^ Interestingly, Xenopus dystroglycan knockdown affected skin morphogenesis via disruption of ciliated precursor cell intercalation; a process also enacted by growth plate chondrocytes in endochondral ossification.^116^ Dystroglycan roles in Notch signalling have been further highlighted by McClenahan et al.^117^ who found that this interaction promoted cell maturation and the formation of a neural stem cell niche in developing mouse brains. In addition, antibody blockade of dystroglycan in neural stem cell neurospheres significantly increased Notch signalling, along with mRNA expression levels of Sox9, a pro‐oligodendrogenic/‐chondrogenic transcription factor.^117^ Moreover, in vitro work validates a satellite cell role for dystroglycan in muscle regeneration, where loss‐of‐function surprisingly resulted in diminished Notch signalling and stem cell dysfunction.^118^ Muscle satellite cell function was also dysregulated in mdx mice, in which lower Notch signalling resulted in less self‐renewal.^119^ Satellite cell loss has also been identified in a rare muscular dystrophy caused by gene mutations in *O*‐glucosyltransferase 1 (POGLUT1, an enzyme that glycosylates extracellular Notch domains) which leads to muscle‐specific α‐dystroglycan hypoglycosylation and aberrant Notch signalling.^120^ This complex role for Notch signalling in muscle has been strengthened by studies in muscles of mdx and dko (dystrophin/ utrophin double knockout) mice and DMD patients which revealed contrasting expression for Notch pathway components.^121^ This complexity has been highlighted in studies where an overexpressed *protective* variant of Jagged1, a Notch ligand, was identified in Golden Retrievers mildly affected by muscular dystrophy; Jagged1 gain‐of‐function was found to ameliorate the dystrophic phenotype in a DMD zebrafish model.^122^ In contrast, Notch pathway over‐activation in dko mice was found to impair muscle regeneration and drive depletion and senescence in muscle progenitor cells.^123^ These data affirm the strong connection between the DGC and Notch pathway signalling (Figure 2).

Notch signalling components are also expressed in emerging skeletal mesenchymal condensations during chondrogenesis in murine and avian limb buds,^124^ where they exert a strong inhibition upon proliferation and differentiation.^125^ Later work underlined these inhibitory roles by showing that selective overexpression of Notch components in the chondrocyte lineage resulted in less cartilage precursor proliferation and less hypertrophic chondrocyte differentiation, culminating in skeletal malformations. In agreement, loss of Notch signalling in this lineage resulted in increased chondrocyte proliferation and hypertrophic zone expansion.^126^ Likewise, retroviral misexpression of Dl‐1 in chick embryo limbs showed that Notch signalling also inhibits chondrocyte maturation by supressing prehypertrophic‐to‐hypertrophic transition to restrict ossification to yield shorter bones^127^; disturbances in Notch signalling likewise regulate the balance between muscle stem cell and progenitor cell populations.^118^ Thus, mouse deficiency models show dominant Notch roles in calvaria and limb primordia where hypertrophic growth plate chondrocytes accumulate to result in shorter bones. With reported exhaustion of mesenchymal progenitors in marrow and an age‐related osteopenia, this suggests that Notch signalling serves to retain mesenchymal progenitors by limiting their differentiation.^128^

Similar Notch pathway‐mediated control of osteoblastogenesis has been verified in MC3T3‐E1 cells, where adenoviral Notch1 (cytoplasmic domain, NCD1) delivery, or activation of endogenous Notch via co‐culture with Dll‐1 expressing myeloma cells, resulted in enhanced bone nodule formation and osteogenic stimulation respectively.^129^ Enhanced mineralisation is also seen in human MSCs (hMSC) transduced with NCD1 or with jagged1, whilst Notch blockade partially delayed osteogenic differentiation.^130^ Notch1 overexpression promotes osteoblast differentiation in hMSCs,^129^ resulting in inhibition of osteoblastogenesis via a suppression of Wnt signalling.^131^ Further studies showed that NCD1 gain‐of‐function limited osteoblast differentiation in ST‐2 and MC3T3‐E1 cells.^132^ Data from transgenic mice with osteoblast‐specific NCD1 overexpression confirmed these Notch pathway roles in vivo by revealing severe osteosclerosis with increased proliferation of osteoblasts^133^; phenotype reversal was attained by osteoblast‐specific RBPjk (recombination signal binding protein for immunoglobulin kappa J) knockout.^134^, ^135^ These data show that Notch activation (Figure 2) exerts cell‐/stage‐dependent control of osteoblast differentiation.^136^

Osteoclasts are likewise controlled via Notch signalling (Figure 2). However, in vitro studies yield conflicting data, with Notch2/Delta‐1 activation driving, and Notch1/jagged1 instead limiting osteoclastogenesis. Inhibition of Notch signalling by a γ‐secretase inhibitor was found to suppress osteoclastogenesis, implying that the stimulatory role of Notch is dominant.^137^ Contrasting data from stromal cells showed that Notch activation limited osteoclast differentiation,^138^ but that Notch2 knockdown in a osteoclast precursor cell line inhibited osteoclastogenesis.^139^ Bone marrow macrophages extracted from mice harbouring an osteoclast lineage‐specific Notch1‐3 deletion underwent more pronounced osteoclastogenesis and precursor proliferation; roles for Notch 1/3 were pinpointed by a lack of detectible Notch2 protein in osteoclasts or their precursors. Notch signalling was found to serve differing roles that were dependent on the stage of osteoclast commitment/differentiation, with activation increasing precursor multinucleation rates and resorptive activity, whilst activation prior to osteoclast precursor induction resulted in reduced osteoclastogenesis.^140^ These studies indicate that osteoclastogenesis and resorptive function are differentially regulated by specific Notch ligand–receptor interactions, directly, in a maturation‐dependent manner. Indirect roles for Notch have also been highlighted, with Notch1 deficient osteoblasts exhibiting elevated RANKL and decreased OPG levels^141^—regulators of osteoclast formation—as well as in murine models of osteoblast‐specific Notch knockdown where an osteoporotic phenotype is observed.^128^, ^133^

Osteocytes similarly exhibit functions for Notch signalling; pathway inactivation using DAPT blocked expression of the early osteocyte marker, DMP1, in IDG‐SW3 cells and led to diminished levels of E11 and dendrite formation as well as spontaneous and disturbed mineral deposition.^142^ This aligns with a role for Notch signalling in osteoblast‐to‐osteocyte transition, which was verified by data revealing Notch‐mediated inhibition of Wnt signalling in late‐stage osteocytes via repression of p‐Akt and the prevention of nuclear aggregation of β‐catenin.^143^ This agrees with the development of osteopetrosis, increased femoral trabecular and cortical bone volume in transgenic mice harbouring osteocyte‐specific Notch activation, and supports roles for osteocyte Notch signalling as a regulator of osteogenesis; intriguingly this also permitted trabecularisation of bone cortices. This phenotype was linked with less resorption and fewer osteoclasts, SOST downregulation and Dkk1 upregulation—consistent with increased Wnt signalling—and reversal of this high mass phenotype upon Notch pathway inactivation further reinforces a somewhat antagonistic relationship between Notch and Wnt in bone.^144^ Increases in bone volume, with abnormal cortical architecture in transgenic mice upon Notch osteocyte‐selective overexpression (Dmp1‐Cre^+/−^;Rosa^Notch^), also support such Notch: Wnt relationships.^145^ Studies by Canalis et al.^146^ indeed demonstrated that Notch deletion in osteocytes led to increased trabecular bone volume with greater osteoblast and fewer osteoclast numbers to reaffirm this relationship.

Fundamental roles for Notch signalling are reinforced by human phenotypes in patients with genetic mutations in Notch signalling components. Alagille syndrome (mutant JAG1) is typified by multiple abnormalities, such as dysfunctional vertebral segmentation (hemivertebrae) and craniofacial abnormalities.^147^ Mutations in Notch2 have also been identified in Alagille syndrome patients (null for JAG1 mutations^148^), further affirming critical Notch signalling roles in the skeleton. Likewise Spondylocostal dysostosis (SCD) patients harbouring mutations in DLL3, MESP2 and LFNG, three genes linked to Notch signalling exhibit short trunk dwarfism due to multiple hemivertebrae and rib defects.^147^, ^149^ These human phenotypes align with those in mice harbouring either the *pudgy* DLL3, a Dll3 targeted,^150^ MESP2 or LFNG gene mutations.^151^, ^152^

Together, these studies identify dystroglycan as pivotal in the Notch signalling pathway, possessing an integral stem cell role as well as a myriad of regulatory roles for Notch in skeletal cells. Similarities in skeletal phenotypes exhibited by MD patients and those harbouring Notch‐associated mutations, in conjunction with signalling disruption during muscle disease, suggests that perturbation in DGC–ECM interactions affect the Notch pathway to ultimately compromise skeletal architecture.

### 4.2 Hippo‐YAP/TAZ signalling

The hippo pathway and its downstream transcriptional co‐factors, YAP (YES‐associated protein) and TAZ (transcriptional coactivator with PDZ‐binding motif) mediate organ size.^153^ In mammals, this pathway consists of a kinase cascade in which MST1/2 phosphorylates its target kinase, LATS1/2, which in turn phosphorylates YAP and TAZ^154^ to prompt their inactivation and thus control gene expression that occurs via the TEAD family of DNA‐binding transcription factors.^155^ YAP/TAZ control cell fate during development and homeostasis in, for example, skin,^156^ lung,^157^ heart,^158^ blood vessels^159^ and brain^160^ by regulating proliferation, differentiation and apoptosis.^161^, ^162^ Control of YAP/TAZ activity via this cascade depends on various inputs, including growth factors,^163^ cell contacts,^164^ as well as membrane^165^ or cytoplasmic sequestration.^166^ Recently, YAP/TAZ were implicated in both the response to local cell mechanics^167^ and in skeletal biology,^168^ and thus, the YAP/TAZ pathway may be vital in mediating its mechanoadaptation.

This is intriguing as Morikawa et al. initially found that the DGC interacted with the Hippo pathway, in their studies in the hearts of Hippo deficient Salv CKO mice. It was also found that Sarcoglycan delta (Sgcd) and Syntrophin B1 (Sntb1), components of the DGC, could serve as downstream YAP targets, with increased Sgcd expression in Hippo deficient cardiomyocytes during regeneration.^169^ Further studies by the same group found that YAP directly bound α‐dystroglycan in the heart, with siRNA knockdown of dystrophin leading to less sarcolemmal/cytoplasmic YAP and its nuclear accumulation. These authors also reported that Hippo phosphorylation of YAP promoted formation of YAP‐DGC complexes in vivo to sequester YAP, thus inhibiting cardiomyocyte proliferation.^170^

Further evidence of this Hippo: DGC connection was found in the decreased YAP activation and reduced focal‐adhesion tensions in myoblasts carrying dystrophin gene missense mutations (those seen in Duchenne and Beckers muscular dystrophy), likely due to a disruption in DGC‐mediated force transmission.^171^ Hippo pathway components and DGC interactions have also been identified in Drosophila, where Kibra and Yorkie (mammalian homologues WWC1/2/3 and YAP/TAZ respectively) were found to interact with dystroglycan in adult fast twitch muscles to control muscle size and integrity.^172^ Role for this YAP‐DGC interaction in mechanotransduction was further evidenced in in vivo contractile loading experiments in mdx mice, where TA muscles possessed intrinsically more YAP protein, were non‐responsive to loading and contained low phosphorylated YAP levels.^173^ This aligns with greater YAP nuclear translocation in mdx muscle and with increased expression of YAP downstream targets. Interestingly, such increases in the expression of focal‐adhesion protein paxillin in mdx muscle, which are themselves consistent with the findings of Sen et al.^174^, led the authors to hypothesise that mdx muscles are under increased cytoskeletal tension, possibly increasing mechanoresponsiveness and contributing to the consistently active YAP signalling.^173^

This strong Hippo pathway: DGC connection justifies an exploration of the roles YAP/TAZ and other pathway components in the control of bone cells and skeletal architecture (Figure 2). It is compelling therefore that YAP/TAZ signalling controls stem cell differentiation during osteogenesis by driving osteoblastic lineage commitment and supressing adipogenesis^175^, ^176^ via ECM‐regulated mechanisms. Thus, conditional Mmp14 deletion in mesenchymal progenitors in vivo was found to redirect skeletal stem cells from osteogenesis to adipogenesis or chondrogenesis via YAP/TAZ.^177^, ^178^ Dupont et al. found that stem cell differentiation was controlled by mechanical stimuli via YAP/TAZ in vitro by showing that osteogenic differentiation of MSCs on a stiff ECM was inhibited by siRNA‐mediated YAP/TAZ silencing. Later studies demonstrated that shear stress‐induced osteoblast differentiation in MSCs was mediated via YAP^179^ and TAZ^180^ nuclear translocation.

YAP/TAZ signalling has likewise been implicated in chondrogenesis by studies in which Yap1 was shown to regulate many stages of chondrocyte differentiation and maturation in vitro and in vivo.^181^ Broadly speaking, YAP is considered a negative regulator of chondrogenesis, as overexpression in hMSC restricts chondrocyte differentiation by blunting sensitivity to BMP.^182^ Likewise, YAP1 overexpression in ATDC5 cells, suppressed chondrogenicity and hypertrophy, and drove proliferation via Wnt/β‐catenin pathway activation.^183^ TAZ has also been shown to play a similar role in studies where Col2a1‐Cre‐driven murine TAZ deletion resulted in skeletal underdevelopment, short growth plates and diminution of all chondrocyte markers. TAZ deletion in chondroprogenitors was found to limit proliferation and maturation in vitro, whilst ectopic TAZ gain‐of‐function promoted both.^184^ Chondrocytes also showed impaired proliferation and maturation upon deletion of Mps one binder adapter protein (Mob1a/b), a hippo pathway factor, which led to mouse chondrodysplasia due to hyperactivation of endogenous YAP1/TAZ and downstream suppression of Sox9.^185^ Recently, Vanyai et al.^186^ demonstrated contrasting results, where a double YAP/TAZ knockout in vitro led to impaired chondrocyte proliferation, yet similar in a cartilage‐specific deficient mouse only led to various skeletal deformities, suggesting that YAP/TAZ is not required for chondrocyte proliferation in vivo but instead controls cartilage morphogenesis via regulation of ECM remodelling. It was recently demonstrated using radial extracorporeal shockwave therapy that YAP/TAZ also served a role in subchondral bone stem/progenitor cell self‐renewal and cartilage repair in vivo.^187^

YAP/TAZ involvement in osteoblastogenesis has also been demonstrated by studies in which YAP was reported to inhibit osteoblast activity by sequestering Runx2.^188^ TAZ, in contrast, was identified as a Runx2 coactivator and PPARγ suppressor thereby driving osteoblastic differentiation of MSCs.^175^ Roles of YAP and TAZ were also were demonstrated in knockout mouse models, where Cre‐mediated deletion of YAP and TAZ, at defined stages in the osteoblast lineage, was used to find that both inhibit full differentiation of osteoblast progenitors.^189^ This contrasted with the effects of YAP and TAZ in mature osteoblasts and osteocytes where both instead promote osteogenesis and inhibit bone resorption.^189^ Their combinatorial activity has also been examined in vivo using allele dose‐dependent ablation of YAP/TAZ which revealed reduced osteoblast activity and bone ECM production, resulting in a lower bone mass and spontaneous perinatal fractures.^190^ Kegelman et al.^191^ found in an adult‐onset‐inducible knockout mouse that later YAP/TAZ deletion expanded the periosteal committed osteoblast precursor pool during callus formation and fracture healing.

Osteocytes, the ‘mechano‐modulatory master regulators’ of bone remodelling, also require YAP and TAZ for the essential mechanotransduction of cell signals to influence downstream gene regulation and activity. For example, the mechanogated ion channel, Piezo1, was shown to control Wnt1 expression and to function as a stimulator of bone formation via YAP1 and TAZ in the MLO‐Y4 osteocyte cell line.^192^ In agreement, osteocyte‐specific conditional YAP/TAZ deletion in mice reduced bone mass and caused dysfunctional ECM production characterised by a disorganised collagen content, thus affecting bone mechanical properties; this coincided with less canalicular remodelling and perilacunar mineral deposition and with lower levels if MMP13‐14 and CTSK expression.^193^ In addition, Zarka et al.^194^ demonstrated the nuclear translocation YAP/TAZ and upregulation of mechanosensitive genes in a 3D osteocyte compression culture model—MLO‐Y4 cells embedded in a collagen hydrogel—in which RNA‐seq analysis revealed that YAP/TAZ knockdown was capable of regulating multiple genes, including those involved in dendrite formation.

Hippo pathway signalling additionally exerts a key role in osteoclast formation and resorption (Figure 2). Thus, ablation of RASSF2 (pathway component) in mice resulted in haematopoietic anomalies, reduced thymus, spleen and BM cell numbers as well as NF‐kB hyperactivation during osteoclast differentiation.^195^ Likewise, MST1 and MST2 are essential for primitive haematopoiesis as embryos lacking both genes formed fewer haemopoietic progenitor colonies in vitro.^196^ MST2‐deficient mice exhibited enhanced osteoclastogenesis characterised by an osteoporotic phenotype, increased osteoclast numbers and NFκB activation in pre‐osteoclasts; RANKL‐induced differentiation was inhibited when MST2 was overexpressed.^197^

The role of Hippo signalling in controlling skeletal cell fate (Figure 2), organ size and in mediating mechanotransduction marks this signalling pathway as central to normal skeletal development and homeostasis, together with known Hippo: DGC interactions and the outcomes arising from muscle disruption suggest perturbation of these interactions within skeletal cells may drive the alterations in skeletal cell behaviour leading to defective skeletal phenotypes associated with muscular dystrophy.

### 4.3 PI3K/AKT signalling

The PI3K/AKT pathway is another signalling cascade controlling a broad range of processes in multiple cell types.^198^, ^199^ PI3K/AKT signalling is initiated by many signals, such as cytokines, growth factors and hormones that activate receptor tyrosine kinases (RTK) and G protein‐coupled receptors (GPCRs), which promote the generation of phospholipids by PI3K. Consequently, effectors, such as AKT and mTORC1 (mammalian target of rapamycin complex 1), are activated downstream of PI3K.^200^ AKT (protein kinase B) is a vital pathway component recruited, with phosphoinositide‐dependent kinase‐1 (PKD1), to the inner surface of the cell membrane, where it phosphorylates and activates AKT, and hence many downstream targets.^201^, ^202^

It is therefore highly pertinent that pathological inhibition of laminin‐dystroglycan binding in muscle cells, decreases AKT activation and induces apoptosis^15^ and that laminin α1 LG4‐5 domain binding to dystroglycan serves to initiate PI3K/AKT signalling.^203^ In corollary, loss of myotube dystrophin (primary DMD characteristic) in vitro prompts enhanced AKT activation, which was also observed in the diaphragm of mdx mice.^204^ PI3K/AKT interaction with DGC was also evident in a canine model of DMD in which deregulated PI3K/AKT signalling with decreased AKT activation was exhibited.^205^ Further reinforcement is observed in dfd13 cells, a dystrophin‐deficient myoblast line cell line derived from a 5‐week‐old mdx mouse, which fail to fully differentiate and which exhibit decreased AKT activation and correspondingly less autophagy.^206^ It is thus interesting that deregulated autophagy is also observed in myotubes derived from an FKRP knockout pluripotent stem cell line, in which 90 genes associated with PI3K/AKT were also shown to be deregulated^206^; patient‐derived FKRP mutant iPS cell lines also showed behaviour indicating defective autophagy.^207^ The intimacy of this PI3K/AKT: DGC interaction has promise in treatment of DMD, with findings in myotubes treated with valproic acid in vitro showing PI3K/AKT pathway activation and less apoptosis and valproic acid‐treated mdx/utrn(−/−) mice showing less muscle fibrosis, inflammatory cells and sarcolemmal damage and increased integrity.^208^

The diverse roles of PI3K/AKT signalling means that it is both essential for cartilage homeostasis and that its mis‐regulation can contribute to osteoarthritis.^209^ ECM composition is vital for cartilage integrity and AKT overexpression is known to enhance proteoglycan synthesis in human chondrocytes under oxidative stress. Furthermore, knockdown of PTEN—a negative regulator of PI3K/AKT and MEK/ERK signalling—increased Col2a1 and aggrecan expression via enhanced phosphorylation of AKT under oxidative stress without any increases in ERK.^210^ PI3K/AKT signalling also functions in ECM catabolism; activation by AKT agonist IGF‐1 producing reduced MMP13 levels to limit rat cartilage injury again via induction of autophagy.^211^ However, studies in rat chondrocytes suggest that PI3K/AKT pathway mainly transduces IGF‐1 effect on Col2a1 expression whilst the MMP13 expression is mediated via the ERK pathway.^212^

The PI3K/AKT pathway has also been implicated in endochondral bone growth (Figure 2). In vitro micromass cultures of embryonic mouse limb bud MSCs and organ cultures of tibiae treated with a PI3K inhibitor showed reduced bone lengthening, with smaller proliferative and hypertrophic zones. This was driven by the inhibition of chondrocyte differentiation, increased apoptosis and reduced levels of hypertrophy.^213^ Its role in chondrogenesis was also described by Kita et al. who found that AKT activation in embryonic limb cultures increased chondrocyte proliferation and hypertrophy, with parallel suppression of RUNX2 and Col10a1 expression. However, inhibition of PI3K was shown to accelerate terminal differentiation but resulted in shorter bones, together suggesting that PI3K/AKT signalling regulates proliferative‐to‐hypertrophic stage transition of chondrocytes.^214^ Studies on the effects of PI3K inhibition in hMSC and articular chondrocyte 3D cultures demonstrated that PI3K/AKT signalling was a stage‐dependent driver of differentiation by upregulating chondroblasts and downregulating differentiated chondrocytes; this study did not report changes in hypertrophy.^215^

PI3K/AKT signalling also regulates osteoblast differentiation in rat models of osteoporosis. PI3K and AKT protein expression was found to be decreased in osteoporotic bone, whilst PI3K inhibition in cultured osteoblasts inhibited osteogenic marker expression, cell proliferation, alkaline phosphatase activity and calcium deposition.^216^ Roles for the PI3K/AKT pathway in bone remodelling via regulation of osteoblastogenesis have been evidenced in MC3T3‐E1 cells cultured in osteoclast‐derived bone resorption supernatant, where osteoblast differentiation/calcification were both accelerated along with an enhanced level of AKT expression, and where AKT knockdown (inhibition of PI3K) reduced calcification and osteocalcin expression.^217^ Similarly, PI3K inhibition in MC3T3‐E1 cells was found to supress osteogenesis, with siRNA knockdown of Akt also resulting in less osteogenesis. These remodelling roles for PI3K/AKT are further supported by findings indicating that both AKT knockdown and PI3K inhibition could influence osteoclastogenesis and osteoclast function^218^ and that mechanical stimulation of MC3T3‐E1 cells activated osteoblast PI3K signalling.^219^

PI3K/AKT pathway involvement in bone development and homeostasis is evident in transgenic mice, such as those deficient in Akt1, which were smaller than WT littermates.^220^ Further studies of 8‐week‐old mice using radiological analyses revealed an osteopenic phenotype with decreased cortical and trabecular bone mass and density, partly attributed to enhanced osteoblast apoptosis rates and suppressed differentiation. Resorptive function was also impaired in these mice due to suppressed osteoclastogenesis, which seem to arise from both direct roles of Akt in osteoclasts and indirect reduction in levels of osteoblast‐derived RANKL.^221^ AKT2‐null mice also exhibit a mild bone growth defect,^222^ which is more severe perinatally in AKT1/2 double knockout mice in which cartilaginous tissue growth is also severely restricted and ossification hardly detected.^223^

Likewise, PTEN knockout (with inherent increases in PI3K/AKT signal), using a col2a1 Cre mouse, resulted in shifts in bone remodelling with increased skeletal size and greater trabecular and cortical bone mass, especially in vertebrae. Growth plates in these mice exhibited disorganised and excessive ECM synthesis with accelerated terminal chondrocyte differentiation.^224^ Targeted PTEN deletion in osteoprogenitors stimulated an increase in progenitor survival and proliferation, yet bones in these mice were shorter and wider due to accelerated osteoblast differentiation, consistent with an uncoupling from its link to chondrocyte differentiation.^225^

In osteocytes, PI3K/AKT signalling is involved in an intracellular mechanotransduction pathway where stimulation causes shear stress to be sensed by dendritic processes, transmitting the signal to the cell body via the PI3K/AKT pathway.^226^ Inhibition of this signalling was shown to induce autophagy in cadmium‐induced bone injury mouse models and in the MLO‐Y4 osteocyte line.^227^ Interestingly, a recent study by Lara‐Castillo et al.^228^ demonstrated that muscle secreted factors were capable of activating PI3K/AKT in MLO‐Y4 cells, suggesting that in addition to mechanical loading, molecular crosstalk between muscle and bone also influences the osteocyte response to load. PI3K/AKT signalling has also been implicated in cell–cell communication in osteocytes where platelet‐derived growth factor‐AA was shown to enhance dendritic process and gap junction formation and therefore cell–cell communication via PI3K/AKT signalling.^229^

PI3K/AKT signalling has been shown to be involved in a broad range of processes within skeletal cell types (Figure 2) and exhibits dysregulation in both in vivo and in vitro models of muscular dystrophy. Overall, this highlights the potential for disruption of DGC–ECM interactions associated with muscular dystrophy to impact PI3K/AKT signalling within skeletal cells, manifesting as skeletal phenotypes observed in MD patients.

### 4.4 ERK: MAPK signalling

The extracellular signal‐related kinase (ERK): mitogen‐activated protein kinase (MAPK) pathway is a widely expressed cascade regulating a multitude of processes, including proliferation, differentiation, migration, metabolism, growth and survival.^230^ ERK/MAPK signalling is essential for development and homeostasis of brain,^231^ nervous system,^232^ muscle,^233^ lungs,^234^ heart^235^ and joints.^236^, ^237^, ^238^ It has been known for some time that dystroglycan binding to laminin‐1/10/11 can block integrin binding and subsequent ERK activation.^84^ Spence et al. identified β‐dystroglycan as a signalling scaffold, interacting with MEK2, the upstream kinase of ERK, in membrane ruffles of HeLa cells. β‐dystroglycan was also shown to interact with ERK directly, within fibroblast focal adhesions, suggesting dystroglycan differentially targets ERK pathway components in a way that is influenced by cell architecture.^239^ This ERK: DGC connection is also evident in the reduced ERK signalling, linked to low cell survival, observed in FKRP knockout and patient‐specific FKRP mutant myotubes in vitro.^207^ Dystroglycan interaction with ERK signalling has also been found in brain injury and scratch‐injury models, where siRNA dystroglycan knockdown in astrocytes decreased mechanical force‐activated ERK signalling thus highlighting direct roles for α‐/β‐dystroglycan.^240^ Intriguingly, IIH6 antibody‐mediated blockade of dystroglycan‐laminin binding revealed that the ERK activation induced by oxygen/glucose deprivation in astrocytes also relied on this DGC binding.^241^

ERK/MAPK signalling is activated by mitogens, cytokines and growth factors (fibroblast growth factors (FGFs),^242^ bone matrix proteins (BMPs)^243^ and transforming growth factors (TGFs)^244^) that activate small RAS‐GTPases which activate RAF. RAF, in turn, phosphorylates MEK, which phosphorylates ERK.^230^ There are two ERK isoforms (1/2) that are activated by MEK1 and MEK2 leading to phosphorylation of membrane, nuclear and cytoskeletal proteins.^245^ This pathway is essential for normal skeletal development and homeostasis. In humans, mutations in upstream MAPK cascade components result in syndromes, such as Cardio‐facio‐cutaneous syndrome,^246^ Costello syndrome^247^ and Noonan syndrome^248^ in which short stature, limb and craniofacial abnormalities arise. Newbern et al.^249^ demonstrated that haplo‐insufficient ERK2 led to conotruncal and craniofacial anomalies comparable to DiGeorge syndrome. Correspondingly, inactivation of ERK2 in osteoprogenitor cells of ERK1 null mice (ERK1^−/−^; ERK2 flox/flox; Prx1‐Cre) caused severe limb/skull deformity with defective mineralisation due to the inhibition of osteoblast differentiation, accelerated chondrocyte terminal differentiation and decreased osteoclastogenesis (Figure 2).

Inactivation of chondrocyte ERK2 in ERK1 null mice (ERK1^−/−^; ERK2 flox/flox; Col2a1‐Cre) led to severe chondrodysplasia, with osteoclastogenesis also inhibited.^250^ Sebastian et al.^251^ found that these mice had longer cartilaginous femoral and humeral elements, suggesting that ERK1 and ERK2 negatively regulate the growth of cartilaginous long bones. Interestingly, the skeletons in ERK1 null mice appear however to develop normally, suggesting that ERK1 is not required.^252^ Osteoblast‐specific expression of MEK1 showed that activated ERK could stimulate osteoblast differentiation by phosphorylating RUNX2 and that dominant‐negative MEK1 resulted in skeletal hypomineralisation.^253^ These links were supported by data from transgenic mice harbouring osteoprogenitor‐specific MEK1/2 (Mek1^Osx^Mek2^−/−^) deletion, which produced severe femoral osteopenia due to delayed endochondral ossification, characterised by an extended hypertrophic zone, impaired osteoclast activity and a reduction in bone formation markers.^254^ This also aligned with the previously implied roles for ERK activation in osteoblast mechanoadaptive responses.^255^, ^256^

ERK signalling has also been shown to regulate osteocytes differentiation where ERK 1/2 inactivation in osteoprogenitors (ERK1^−/−^; ERK2 flox/flox; Prx1‐Cre) resulted in robust decreases of Dmp1 and osteocytes lacking dendrites and the characteristic lacunar‐canalicular system.^257^ These data demonstrate essential roles for ERK signalling in multiple aspects of skeletal development, where its disruption results in overt skeletal phenotypes (Figure 2). The intimate relationships that we have highlighted to exist between ERK signalling and both skeletal integrity and DGC function positions the phenotypic characteristics of MD patients in a clear skeletocentric focus (Figure 2) and suggest that disrupted DGC interactions may precipitate a defective range of downstream signalling in skeletal cell types that culminate in bone failure in these patients.

### 4.5 Src signalling

Src, a non‐receptor tyrosine kinase member of the Src family kinases, is involved in controlling a wide array of cell activities, including transcription, adhesion, differentiation, proliferation and apoptosis.^258^ It is therefore highly relevant that laminin binding by α‐dystroglycan leads to Src‐mediated phosphorylation of β‐dystroglycan.^259^ Studies by Sotgia et al.^260^ identified that Src, and other SH2 domain containing proteins, induced tyrosine phosphorylation of β‐dystroglycan leading to a disruption in its interaction with utrophin^259^ and dystrophin.^261^ Src‐dependent phosphorylation of β‐dystroglycan was also pinpointed in studies showing that overexpression of β‐dystroglycan blocked podosome formation in myoblasts by Tks5 and Src sequestration into a ternary complex; mutations in the dystroglycan phosphorylation site restored podosome formation.^262^

These Src: DGC links are bolstered by data showing that Src is highly expressed in mdx mouse muscle, contributing to the dystrophic phenotype.^263^ In the absence of dystrophin, β‐dystroglycan is exposed and consequently phosphorylated by Src leading to its proteasomal destruction.^264^ By crossing mdx with mice harbouring knock‐in of a phenylalanine substitution at a key tyrosine dystroglycan phosphorylation site, Miller et al.^265^ demonstrated that preventing phosphorylation of dystroglycan ameliorates the dystrophic mdx phenotype. A similar strategy, targeting the phosphorylation of β‐dystroglycan through the use of Src tyrosine kinase inhibitors, was used by Sanarica et al.^266^ in mdx mouse muscle where, despite β‐dystroglycan levels being restored and phosphorylation reduced, they found only modest improvement in functional parameters.

Src has also been identified as important in bone homeostasis (Figure 2); overt osteopetrosis has been observed in Src‐deficient mice and aberrant Src signalling is linked with bone metastases.^267^ It is therefore compelling that osteoclasts possess surface podosome structures, which enable ECM binding; they are found on the ventral surface of osteoclast precursors, where columnar actin puncta develop^268^ to form peripheral adhesions that promote efficient bone attachments. During the early phases of osteoclast differentiation, these podosomes organise into rings to allow for both the cell fusion required for multinucleation and to facilitate migration during cycles of resorption.^269^ It is therefore somewhat surprising that the number of osteoclasts in Src‐deficient mice was raised compared with wild‐type mice, but that these osteoclasts failed to form resorption pits, indicating that Src does not affect osteoclast differentiation but is required for bone resorption.^270^

Src also serves a role in osteoblasts, with greater osteoblast activity and an osteopetrotic phenotype (and, as expected, less resorption) in Src knockout mice (Figure 2). In vitro studies showed that ALP activity was increased in Src^−/−^ osteoblasts, suggesting Src inhibits osteoblast differentiation.^271^ This aligns with data showing that Src inhibits Runx2 by promoting YAP binding to suppressnuclear translocation. However, Src inhibition was shown to block YAP phosphorylation and to dissociate Runx2–YAP complexes.^188^ In contrast, Src also promotes osteoblast differentiation by binding and phosphorylating osterix, initiating its nuclear translocation^272^ to drive differentiation.

Overall, these studies demonstrate that DGC–ECM interactions foster Src: DGC engagement and are crucial to muscle development and serve roles in both osteoblast differentiation and osteoclast podosome‐dependent resorption to result in the skeletal phenotypes exhibited in Src‐deficient mice, together suggesting a role in skeletal cells in dystrophic bone phenotypes (Figure 2).

## 5 NEW INSIGHTS

The multiple roles of the aforementioned three ECM dystroglycan ligands in the skeleton, and the various skeletal phenotypes exhibited upon manipulation of the intracellular signalling pathways that function downstream of DGC engagement, are supportive of *direct* DGC inputs to skeletal tissue dynamics. The skeletal phenotypes in muscular dystrophy patients and mouse models of this harmful disease have, nonetheless, been almost entirely explained from a *musculocentric* viewpoint, asserting that their aetiology is solely due to muscle dysfunction. To test this more directly, skeletal architecture should be more fully evaluated in mouse muscular dystrophy models where DGC function is modified in distinct ways, which nonetheless yield similar muscle deficits (e.g. mdx and FKRP knockdown). These analyses should examine if phenotypes indeed align, and they should be performed in very young mice that are overtly normal in terms of assessed muscle function. It would also be beneficial if, unlike earlier studies which examined only isolated bone regions, these analyses were applied to explore changes in bone architecture across entire long bones (and skulls) to more fully capture any instructive anomalies in skeletal architecture (manuscript in preparation).

If such studies in mdx and FKRP knockdown mice were to reveal modifications in bone organisation before onset of overt muscle damage, and conceivably highlight divergent trabecular and cortical bone architecture, they would imply that the bone phenotypes in both, or at least one of these mutants arises via *skeletocentric* origins. At the very least, non‐identical phenotypes would point to their emergence as a product of an interplay between the direct DGC roles in muscle and skeletal cells.^273^, ^274^ Thorough skeletal examination in young mdx mice would extend the work by Nakagaki et al.^40^ where deficits in bone mass were found in small bone segments of male 3 week‐old mdx mice. Alternatively, they may confirm the findings of Gao et al.^41^ and Wood et al.^42^ which reported greater cortical mass in mdx mice. To date, bone has not been examined in FKRP knockdown mice; should the changes in these mice not fully mirror the architectures seen in similarly aged mdx mice, this would imply that functional DGC engagement is essential in the emergence of skeletal form, independently of consequences that might arise via muscle dysfunction.

Muscle action is known to also influence the endochondral ossification process that is responsible in controlling bone length.^275^, ^276^, ^277^ Evaluation of tibia and femur bone length in young mdx mice is however needed to more fully explore reports of accelerated longitudinal growth.^41^, ^42^ Divergent bone length in FKRP knockdown mice would likewise support DGC roles in endochondral ossification, independently of muscle dysfunction. Moreover, histological analysis of growth plate zones in very young mdx and FKRP knockdown mice would assist in identifying whether DGC exerts predominant roles in proliferating and/or hypertrophic zone function. These studies may also reveal the likely cellular mechanisms involved, for example presence of increased proliferation and hypertrophy in mdx mice would align with increased AKT activation in these zones.^214^ Greater AKT activation was indeed seen in dystrophin‐deficient diaphragm muscle and primary myotubes in vitro.^204^ No studies, to date, have sought to resolve bone length in FKRP knockdown mice, and thus, the functional role of DGC: ECM engagement in skeletal growth and maturation remains undefined.

Interrogation of skull morphology may also disclose clues to the skeletal roles of DGC engagement. Comparing skull bones in mdx and FKRP mutants, possessing hypo‐glycosylated dystroglycan and hence reduced DGC‐ligand deposition and binding,^278^, ^279^, ^280^, ^281^ would highlight any conserved role for *direct* DGC inputs to skeletal dynamics. Studies to identify the skeletal cell targets for DGC inputs would also benefit from in vitro studies that, for instance, exploit IIH6 antibody‐mediated blockade of α‐dystroglycan binding to LG‐domain‐containing ECM ligands. These could be applied to a range of cell types, including bone marrow‐derived osteoclasts, primary osteoblasts and chondroprogenitor ATDC5 cells in which functional endpoints could be monitored to test whether the reported functions of DGC binding in regulating skeletal cell behaviour during endochondral ossification and bone re/modelling are indeed substantiated.^282^, ^283^

These studies would extend the wealth of prior data demonstrating the effects of dystrophin dysfunction—a presumed consequence solely of muscle failure—with marked differences in microarchitecture, static and dynamic histomorphometry, biomechanics and gene expression in bone in mdx and mdx/utn (utrophin) double knockout (dko) mice. In general, mdx and dko mice exhibit deficits in trabecular and cortical mass and density when examined in defined bone regions.^37^, ^38^, ^40^, ^41^, ^284^, ^285^, ^286^ The likelihood that alternative mechanisms explain this bone frailty is evident in studies where human osteoblasts from healthy donors were treated with DMD patient sera (or wild‐type mouse calvariae were cultured with mdx mouse sera), which showed reduced mineralisation, downregulation of osteogenic and upregulation of pro‐osteoclastogenic cytokine mRNAs; the blunting of these changes by co‐incubation with an IL‐6‐neutralising antibody suggest IL‐6 is a vital bone loss mediator in DMD.^37^ The possibility that the skeletal changes in dystrophic mice may not solely be a consequence of altered muscle function is also evident in the spectrum of degenerative changes in bone, articular cartilage and intervertebral discs as well as a reduced capacity for bone healing observed in dko mice.^38^ Segregation of muscle/bone phenotypes is also observed in functional tests, where tibial bone in young mdx and dko mice was less impaired than muscle, prompting speculation that the elucidation of other contributing factors to bone strength and function is necessary.^282^, ^283^ Indeed, parabiosis studies suggest that blood‐borne factors and/or progenitors may be partly responsible for the bone deficiencies that emerge in dKO mice.^286^

Intriguingly, a recent study that evaluated the entire cortical compartment of tibiae from mdx and dko mice surprisingly found increases in microarchitectural parameters in specific locations that do not entirely align with a reduced skeletal fracture resistance.^287^ Likewise, although modifying muscle contractile activity by low‐frequency stimulation did not change bone shape in healthy mice, it nonetheless engendered cortical bone thinning and reduced mechanical strength in mdx and dko mice, leading to hypotheses that bone in these dystrophic models exhibits greater sensitivity to contractile activity, even though it has been concluded that muscle pathology occurs earlier than bone pathology in these mice.^284^

The effects of glucocorticoids on bone parameters in mdx and dko mice have also been evaluated to fully discern the mechanisms underpinning the deleterious skeletal effects in human patients.^288^, ^289^, ^290^, ^291^ Growth hormone treatment protected against glucocorticoid‐related bone loss, yet seemingly without bone microarchitectural changes.^287^ Such studies have led to potential bisphosphonate^292^ and anti‐RANKL therapies^293^ to protect against glucocorticoid‐induced bone loss which, intriguingly, also seemingly improve muscle function. The use of mice at ages when muscle function is reported to be compromised by degeneration and/or glucocorticoid treatment make it difficult, however, to separate direct and indirect DGC roles in dystrophic skeletal phenotypes.

This review highlights the multiplicity of complex and varied ECM interplay and downstream signalling pathway targets, via which DGC roles can be controlled to regulate skeletal architecture and mass. Elucidation of the underpinning molecular mechanisms will be complex but it is crucial that they are fully clarified. It is clear the multitude of ECM: DGC interactions and their downstream signalling pathways are also pertinent within osteochondral cells to regulate the development and homeostasis of the skeleton. Historically, the skeletal phenotypes associated with muscular dystrophy have been attributed to the lack of muscle‐induced loading and for the most part any direct roles for the DGC in skeletal cell function may have been overlooked. Only a handful of studies have investigated skeletal phenotypes in mouse DMD models, and the cellular and molecular mechanisms driving such changes in the many muscular dystrophy subtypes have yet to be fully explored. Illumination of the plethora of DGC roles that potentially impact skeletal cell types will likely identify novel therapeutic targets, and they also imply that it may be beneficial if treatment strategies for MD are also simultaneously useful in targeting the skeleton.

## FUNDING INFORMATION

This review is invited in response to the Fell‐Muir Award given to AP by the British Society for Matrix Biology. The author gratefully acknowledges the excellent work of Mark Hopkinson as part of his PhD studies. We are grateful for financial support received during this time from the Medical Research Council UK, Versus Arthritis and an MRC ImagingBioPro Network Proof of Concept Award.

## 6 CONFLICT OF INTEREST STATEMENT

The authors declare no conflicts of interest.
